# A PID-Based kNN Query Processing Algorithm for Spatial Data

**DOI:** 10.3390/s22197651

**Published:** 2022-10-09

**Authors:** Baiyou Qiao, Ling Ma, Linlin Chen, Bing Hu

**Affiliations:** School of Computer Science and Engineering, Northeastern University, Shenyang 110819, China

**Keywords:** kNN query, PID, spatial big data, density peak clustering, Spark

## Abstract

As a popular spatial operation, the k-Nearest Neighbors (kNN) query is widely used in various spatial application systems. How to efficiently process a kNN query on spatial big data has always been an important research topic in the field of spatial data management. The centralized solutions are not suitable for spatial big data due to their poor scalability, while the existing distributed solutions are not efficient enough to meet the high real-time requirements of some spatial applications. Therefore, we introduce the Proportional Integral Derivative (PID) control technology into kNN query processing and propose a PID-based kNN query processing algorithm (PIDKNN) for spatial big data based on Spark. In this algorithm, the whole data space is divided into grid cells of the same size using the grid partition method, and the grid-based index is constructed. On this basis, the grid-based density peak clustering algorithm is used to cluster spatial data, and the corresponding PID parameters are set for each cluster. When performing kNN queries, the PID algorithm is used to estimate the radius growth step size of kNN queries, thereby realizing kNN query processing with a variable query radius growth step based on a feedback mechanism, which greatly improves the efficiency of kNN query processing. A series of experimental results show that the PIDKNN algorithm has good performance and scalability and is superior to the existing parallel kNN query processing methods.

## 1. Introduction

kNN query is a very important spatial operation and is defined as: given a set of spatial objects and a query point, find the *k*-nearest spatial objects to the query point. For example, find the five restaurants closest to the school. kNN query is widely used in various spatial application systems such as geospatial information systems, location service systems, ocean and atmosphere information service systems, etc. In recent years, with the rapid development and wide application of the Internet of Things, earth observation technology, and location-based service technology, the size of spatial data has increased dramatically, and spatial data has become a very important type of big data. Because of the huge data size of spatial big data, how to efficiently process kNN queries on spatial big data has become a challenging problem. It has always been one of the research hot spots in the field of spatial data management. The traditional centralized solutions are not suitable for kNN query processing on spatial big data due to their poor scalability. The existing distributed solutions mainly include MapReduce-based algorithms [1,2,3] and Spark-based algorithms [4,5,6,7]. Since MapReduce is a disk-based parallel processing framework, it cannot efficiently support iterative processing, so it is not suitable for online query processing. As an in-memory computing framework, Spark has a faster processing speed than MapReduce. At present, there are some big data processing systems based on Spark, such as Geospark [4], a cluster computing framework for geographic data, Simba [6], a big data analysis system based on Spark SQL, SparkNN [7], the latest kNN query processing method based on KD-tree, etc. The Spark-based solutions can be simply divided into tree index-based methods and grid index-based methods. The tree index-based methods include R-tree-based methods, KD-tree-based methods, etc. These methods first find the spatial location of the query point through the index and then perform a kNN query by continuously expanding the search range. The query processes of these methods are relatively complicated, which affects the query processing efficiency. Compared with the tree index-based methods, the grid index-based methods, such as ParallelCircleTrip, etc., have simple structures, fast data positioning, and lower maintenance cost. Therefore, most of the existing distributed kNN query processing methods use grid index or grid-based multi-level index structure. However, most of the existing kNN query processing algorithms based on grid index use a fixed-step query radius expansion method, which requires more time to expand the query radius and scan the data during query processing, thus affecting the efficiency of kNN query processing. 

Aiming at the problems of the existing methods, we introduce the Proportional–Integral–Derivative (PID) control technology into kNN query processing and propose a PID-based kNN query processing algorithm (PIDKNN). In this algorithm, we introduce PID controller technology into the calculation of the kNN query radius growth step and combine grid indexing and density peak clustering method to realize the kNN query processing with variable query radius growth step based on a feedback mechanism, which greatly improves the efficiency of spatial kNN query processing. The main contributions of this paper are as follows:

- We introduce the PID controller technology to the calculation of the kNN query radius growth step and realize the kNN query processing with a variable query radius growth step. This is the first time that the PID controller technique is introduced into the kNN query processing, making full use of the feedback mechanism of the PID controller, significantly improving the efficiency of spatial kNN query processing.
- We use the grid partitioning method and row sorting encoding to realize the partitioning and encoding of spatial data and construct a grid-based data index, which fully utilizes the advantages of simple structure and fast positioning of the grid index. We used a grid-based density peak clustering algorithm to optimize the setting of PID parameters and further improve the processing efficiency of spatial kNN queries.
- We conduct extensive performance evaluations using real and synthetic datasets. A series of experimental results show that our PIDKNN algorithm has good query processing performance and scalability and outperforms the existing kNN query algorithms.

## 2. Related Work

kNN query has always been an important research topic in the field of spatial data management, and there has been a lot of research work at present. For centralized kNN query processing, researchers put forward a series of kNN query processing algorithms, including grid index-based kNN query algorithm [8,9], tree index-based kNN query algorithm [10,11,12,13], Voronoi diagram-based algorithm [14,15], moving object-oriented continuous query processing [16], kNN query algorithms for new GPU hardware [17,18,19], etc. Since the focus of this paper is the kNN query algorithm in a distributed environment, the following will focus on the research progress of distributed kNN query processing algorithms. 

In terms of distributed kNN query processing, a parallel kNN joint query processing algorithm, pgi-distance [20], was proposed, which uses grid and B+ tree double-layer index structure to perform parallel kNN query processing with a fixed radius growth step. For continuous kNN queries on moving objects, Bareche [21] proposed a distributed hybrid index VS-TIMO and a corresponding query processing algorithm in which moving objects are classified and partitioned according to the speed and indexed by using VS-TIMO; it has high query processing efficiency. Yang [22] proposed a distributed kNN query algorithm for moving objects. The algorithm adopts a two-layer grid index, the top layer is a grid structure, and the bottom layer is composed of multiple blocks. A block corresponds to one or more grids at the top level. It has good query processing efficiency. In recent years, with the rapid development of cloud computing and big data technology, researchers have proposed a series of kNN query algorithms based on distributed computing frameworks such as MapReduce and Spark. Zhang [1] proposed a kNN query algorithm, using space-filling curves and R-tree to index spatial data, achieving kNN approximate query processing based on a MapReduce framework. Bagui [2] proposed a MapReduce-based parallel kNN algorithm. The algorithm uses local sensitive hash to preprocess the data, greatly reduces the calculation time, and improves the efficiency of the kNN query. ParallelCircleTrip [3] is a MapReduce-based implementation of the traditional CircleTrip algorithm. This method uses grid index to improve the kNN query processing efficiency, but still uses a fixed query radius growth step. Jang [23] uses a dynamic grid index to prune unnecessary grid cells through distance-based filtering, thus reducing data transmission and computing overhead. To a certain extent, the efficiency of the query is improved, but the algorithm still uses a fixed radius growth step to change the query range. 

Because Hadoop MapReduce is a disk-based distributed computing framework, its response time is relatively slow, so it is not suitable for online queries. Therefore, more and more researchers have proposed spatial query processing algorithms based on the Spark framework [4,5,6,7,24,25,26,27,28], which is an in-memory computing framework with a faster processing speed. Xie [6] proposed a spatial big data analysis system based on Spark, which expanded the Spark SQL engine and supported the construction of an RDD-based memory index, thus effectively supporting various query operations such as range query and kNN query. This method uses the global index to get a large kNN candidate set and then narrows the scope to obtain accurate kNN query results. Aghbari [7] proposed a spatial data-oriented memory partitioning and indexing system, SparkNN, based on the Spark framework. The system consists of three layers: data partitioning layer, local index layer, and global index layer, and adopts the KD-tree index. Compared with the original Spark, SparkNN significantly improves the average query time. Moutafis [26] proposed the first distributed GKNN query algorithm in Apache Spark, and this method proved to be more efficient than Apache Hadoop.

PID controller has been widely used in process control as a negative feedback control system to keep the system stable. It can be understood as a controller that considers present, past, and future errors [29]. Fractional-Order PID (FOPID) is the expansion of the conventional PID controller based on fractional calculus. The FOPID controller has better robustness and is more flexible. However, the conventional FOPID controller tuning methods are less efficient and cannot reach the global optimum. Amirahmadi [30] used SPEA multi-objective optimization to overcome the difficulties in designing FOPID controllers. SPEA was shown to provide effective regulation of both PID and FOPID controllers. Wan [31] proposed a fractional-order PID using a cloud-model-based genetic algorithm (CQGA), which is more effective in tuning the parameters of the FOPID controller compared to the genetic algorithm. Shalaby [32] proposed a machine learning-based method for online tuning of FOPID controller parameters that effectively addresses the effects of parameter uncertainty and disturbances in uncertain nonlinear systems. In this paper, we introduce the PID algorithm into kNN query processing and propose a variable query step kNN query processing algorithm based on the PID feedback mechanism. Since the focus of this paper is on kNN query processing, considering the implementation of existing PID models and the complexity of parameter adjustment and training, we do not use the latest PID control algorithm, but use the traditional PID controller algorithm to realize the calculation of kNN query step size.

## 3. Methods

### 3.1. The Definition of Space kNN Query

As a common spatial query operation, a kNN query usually refers to a given spatial dataset *R*, query point *q*, and returns *k* data objects closest to query point *q* in *R*. The kNN query results can be expressed as $KSet\left(k\right)=\left\{r_{1},r_{2},r_{3},r_{4},\ldots ,r_{k}\right\},\;\left(r_{i}\in R,\;1\leq i\leq k\right),$ which satisfies the following two properties:

(1) Data objects in *KSet(k)* satisfy the inequality $dist\left(q,r_{1}\right)\leq dist\left(q,r_{2}\right)\leq dist\left(q,r_{3}\right)\leq \ldots <dist\left(q,r_{k}\right)$, where $dist\left(q,r_{i}\right)\left(1\leq i\leq k\right)$ represents the distance from query point *q* to the data object $r_{i}$.
(2) For any data object p∈R\KSet(k), satisfies $dist\left(q,p\right)>dist\left(q,r_{k}\right)$.

For kNN queries, the existing tree index-based algorithms usually first determine the location of query points through the index and then expand the search range by backtracking the index until *k* results closest to the query points are found. The grid index-based processing methods usually first find the grid cell where the query point is located according to the grid index and then execute the range query with the query point as the center and the grid cell length L as the radius. If *k* data objects closest to the query point are found, the query will be stopped. Otherwise, the query radius is increased with a fixed radius growth step L and continues the next range search until the conditions are met. Obviously, the process of tree index-based algorithms is relatively complex, while the radius growth step of grid index-based methods is fixed, resulting in more search times, which affects the efficiency of query processing. As a result, we propose a PID-based parallel kNN query processing algorithm for spatial big data based on Spark. Combining grid indexing and PID controller technology, we efficiently achieve kNN query processing with variable query radius growth step, greatly improving the efficiency of spatial kNN query processing.

### 3.2. PID-Based Parallel kNN Query Processing Algorithm

In this paper, we propose a PID-based parallel kNN query processing algorithm based on the Spark framework, which aims to speed up kNN query processing from the perspective of reducing query search times. The algorithm mainly includes three parts: grid index construction, PID-based query radius growth step calculation and PID parameter setting, and PID-based kNN query processing. They are described in detail below.

#### 3.2.1. Grid Index Construction

Compared with the tree-based index, the grid index has the advantages of simple structure, fast data positioning, and lower maintenance cost, so we use the grid partition method to partition the data space and index the spatial data. Firstly, the data space composed of longitude and latitude is divided into grid cells of equal size by using the grid partition method. Each grid cell may be represented by a tuple *c*(*m, n*), where *m* and *n* are the row and column of the grid cell, respectively. Each grid cell is encoded by the row sorting method. Compared with space-filling curves such as Z-order and Hilbert, the calculation of row sorting is relatively simple, and the geographic location information can be quickly located. During the query process, it is more efficient to directly determine whether the grid cell is within the query radius by the row and column where it is located. If the number of grid cells per row is *Row_num_*, the encoded value of grid cell *c*(*m, n*) is calculated by Equation (1):

*c*(*m, n*) = *n* * *Row_num_* + *m* (1)

For each spatial data object $p_{i}$, its spatial position is represented by a two-tuple $\left(x_{i},y_{i}\right)$, $x_{i}$ and $y_{i}$ are the longitude and latitude where $p_{i}$ locates. The data object $p_{i}$ is mapped to the grid cell $c\left(m_{i},n_{i}\right)$, where $m_{i}=⎣x_{i}/l⎦$, $n_{i}=⎣y_{i}/l⎦$, *l* is the length of the grid cell. If *s* data objects are mapped to the same grid cell $c\left(m_{i},n_{i}\right)$, then they can be represented by the set $P\left(m_{i},n_{i}\right)=\{p_{1},\;p_{2},p_{3},\ldots ,p_{s}$}. Each grid cell and all data objects mapped to the grid cell generate an index item. Each index item is organized in the form (Key, Value) pair, where Key is the grid cell code, and Value is a set of all data objects mapped to the grid cell and the number of data objects. For example, the grid cell $c\left(m_{i},n_{i}\right)$ and its dataset $P\left(m_{i},n_{i}\right)=\{p_{1},\;p_{2},p_{3},\ldots ,p_{s}$}, the index item $\left(c\left(m_{i},n_{i}\right),\;\left\{Num,p_{1},\;p_{2},p_{3},\ldots ,p_{s}\right\}\right)$ will be formed, where Num is the number of data objects. All the index items together form a grid index, which is stored in multiple data partitions in the order of Key. When the query is executed, the grid index is loaded into the memory in the form of RDD, which is processed by multiple executors. In kNN query processing, the grid index can be used to quickly locate the grid cell where the query point is located, and the value of Num can be used to determine whether to scan each data object in the grid cell, thus reducing unnecessary computing operations.

**Figure 1:**
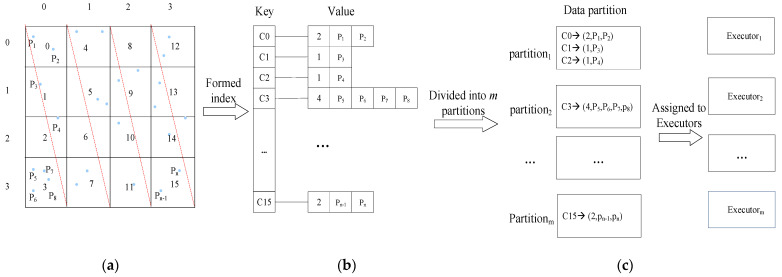
An example of grid index. (**a**) Data space partition; (**b**) grid index; (**c**) index data partition.

Figure 1 is an example of a grid index. In Figure 1a, the whole data space is divided into 16 grid cells, and the grid cells are encoded by the row sorting method. Data objects are mapped to each grid cell according to their spatial positions. Figure 1b is the grid index corresponding to Figure 1a. Grid index and data objects are divided into multiple data partitions, which are stored in different data nodes. When a query is executed, multiple executors perform parallel query processing on partitions, as shown in Figure 1c.

#### 3.2.2. Calculation of PID-Based Query Radius Growth Step and PID Parameter Setting

In this paper, PID control technology is used to estimate the appropriate kNN query radius growth step. Through the negative feedback adjustment mechanism of PID, the search radius can be dynamically adjusted, thus reducing the kNN search times and improving the efficiency of kNN query processing. The calculation equation of the kNN query radius growth step based on PID and the setting method of PID parameters will be given.

1. Calculation of query radius growth step based on PID

**Figure 2:**
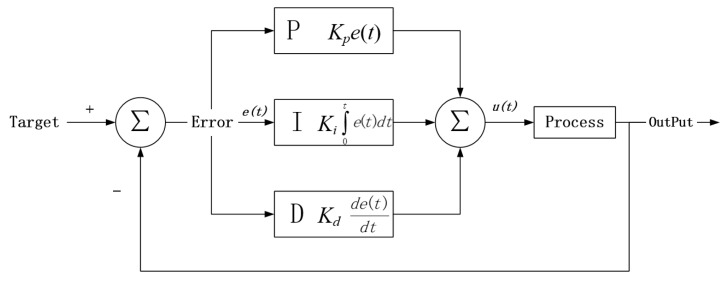
Structure of PID system.

As an automatic control algorithm widely used in industry, the PID control algorithm has the characteristics of simple principle, easy realization, and wide application range. It can automatically adjust the output according to the error between the system output and the target and make the output consistent with the target. The structure of the PID system is shown in Figure 2.

In Figure 2, the input is *Target*, representing the target value of the system, and the *output* is the output value of the system. The PID algorithm mainly consists of three parts: proportional module P, integral module I, and derivative module D. The P module directly adjusts the output to improve the response speed of the system. The I module is used to correct the error generated by the system to improve the control accuracy. Both P and I modules are adjusted after errors are generated. Module D belongs to advance control, which will bias the output in the opposite direction of the proportional and integral to hopefully prevent the controller from overshooting. It can be seen from Figure 2 that the PID control process is relatively simple. When the target value is input, the system calculates its predicted value *u*(*t*) according to the PID calculation equation and obtains the difference value *e*(*t*) between the target and *u*(*t*). Then, it is fed back to the PID system, *u*(*t*) and *e*(*t*) are recalculated, and the calculation process is iterated until *e*(*t*) falls within the PID system’s error range. The calculation method of the PID control system is shown in Equation (2):

(2) $$u\left(t\right)=K_{p}e\left(t\right)+K_{i}\int_{0}^{t}e\left(t\right)d_{t\;}+K_{d}\frac{de\left(t\right)}{dt}$$

where *e*(*t*) is the difference between the PID system’s output and input values, *t* is the time, $K_{p}$ is the parameter of module P, $K_{i}$ is the parameter of module I, and $K_{d}$ is the parameter of module D.

To facilitate the calculation of the kNN query radius size, Equation (3) is discretized. According to the principles of calculus, the integral module I and derivative module D in the system are approximated by discrete values, and the derivative part is replaced by the difference part so as to obtain the calculation of the kNN query radius growth step. See Equation (3):

(3) $$u\left(s\right)=K_{p}e\left(s\right)+K_{i}\sum_{j=0}^{s}e\left(j\right)+K_{d}\left[e\left(s\right)-e\left(s-1\right)\right]$$

where u(s) is the predicted query radius growth step, *s* stands for the *s*-th kNN query processing (*s* = 0, 1, 2, …), e(s) is the difference between the preset kNN query result number *k* and the actual number of query results obtained from the previous *s* kNN query processing.

The error e(s) can be calculated according to the number of the current kNN query results and the kNN query target value *k*. Then, according to the PID calculation equation mentioned above, the increment step of the kNN query radius is calculated to realize the kNN query with the variable query radius growth step. Obviously, by setting three parameters, *K_p_*, *K_i_*, and *K_d_*, reasonably, we can achieve the prediction of the kNN query radius growth step quickly, accurately, and stably.

2. PID parameter setting

It can be seen from Equation (3) that the values of the three PID parameters *K_p_*, *K_i_*, and *K_d_* directly affect the calculation of query radius step size and determine the efficiency of the kNN query processing, so the values of the three parameters are very important. The values of the three parameters are related to the distribution of data, the position of query points, and the size of the *K* value. For this reason, we use a grid-based density-peak clustering algorithm to cluster grids of different densities into different clusters and set the respective PID parameters for each cluster. By setting and training different PID parameters for each cluster, we can solve the problem of query efficiency degradation due to data skewing to a certain extent and improve the query processing efficiency in general. The density peak clustering algorithm uses the number of data objects in each grid cell as the grid density and is able to automatically find the center of clustering, thus achieving efficient clustering of grid cells. Compared with other clustering algorithms such as k-means, the density peak clustering algorithm is simple, efficient, and more suitable for grid indexing. The specific methods are as follows:

(1) The density peak clustering algorithm is used to cluster the grid cells, and the whole data space is divided into different categories according to the clustering results. The specific steps of density peak clustering are as follows:

Step 1 determines the local density. For each grid cell *i*, its local density $\rho_{i}$ is determined by the number of data objects mapped into the grid cell.

Step 2 determines the relative distance. In this paper, the distance between grid cells is defined as the Euclidean distance between their center points. For each grid cell *i*, its relative distance *δi* is measured by computing the minimum distance between grid cell *i* and any other grid cell with higher local density. *δi* can be expressed by Equation (4):

(4) $$\delta i=\underset{j:\rho_{j}>\rho_{i}}{\min}\left(D_{ij}\right)$$

where $D_{ij}$ is the distance between grid cell *i* and *j*. If the grid cell *i* has the highest local density, its relative distance δi is defined as:

(5) $$\delta i=\underset{i\neq j}{\max}\left(D_{ij}\right)$$

For the grid cell with the highest local density, that means the density of the grid cell is greater than or equal to that of any other grid cell. Therefore, the algorithm assumes that this grid cell must be the density peak and artificially sets its relative distance as the maximum, as shown in Equation (5).

Step 3 performs density peak clustering. The grid cells are sorted according to their local density and relative distance in descending order, and multiple top grid cells are selected as the density peak points. All grid cells are clustered according to the density peak clustering algorithm to form multiple clusters centered on the density peak.

(2) For each cluster, *m* kNN queries with different *k* values are created at random. PID parameters are set in the way of proportion, integration, and differentiation, and the PIDKNN query processing algorithm is executed (see Section 4.3 for details) to determine the optimal PID parameters according to the execution results. The methods are as follows:

First is the determination of the *K_p_* value. The *K_p_* value is initially 0, and then gradually increases. For each *K_p_* value, *m* kNN queries are executed to obtain the average execution time *T* and the average number *N* of the candidate results generated by these queries. The *K_p_* value with the minimum average query time *T* is preferentially selected as the *K_p_* parameter value of the cluster for this *k* value. When the query time *Ts* of a different *K_p_* value is the same, the *K_p_* value with a smaller number *N* of candidate result sets generated by these queries is selected as the *K_p_* parameter value of the cluster for this *k* value.

Second, on the basis of the determined *K_p_*, we use the same method to determine *K_i_*. Starting from 0, we increase the value of *K_i_* and select the *K_i_* value with the shortest average query time *T* as the candidate parameter value of *K_i_*. The differential coefficient *K_d_* is determined by the same method as described above.

**Figure 3:**
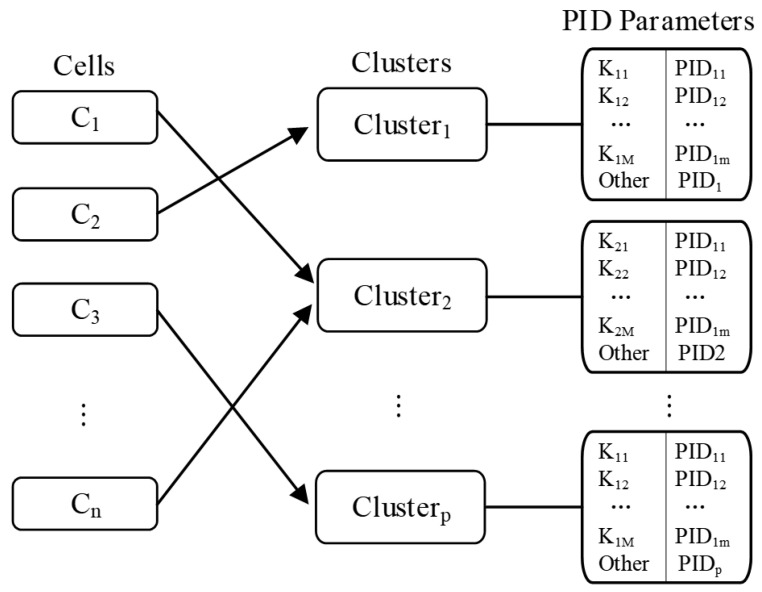
Mapping of grid cells, clusters, and PID parameters.

(3) A PID parameter table is used to store the optimal PID parameters for each cluster under different *k* values. Each grid cell and its cluster, together with the corresponding PID parameters, are saved in the PID parameter table. The mapping relationship is shown in Figure 3. Among them, *C_1_*, *C_2_*, …, *C_n_* represent grid cells, which are divided into different clusters through the density peak clustering algorithm. Each cluster corresponds to a PID parameter table, which stores PID parameters under different *k* values. When a kNN query is run, the grid cell where the query point is located can be obtained, and the clustered grid cells can be found in the PID parameter table to find the PID parameters corresponding to the *k* value.

#### 3.2.3. PIDKNN Algorithm

**Figure 4:**
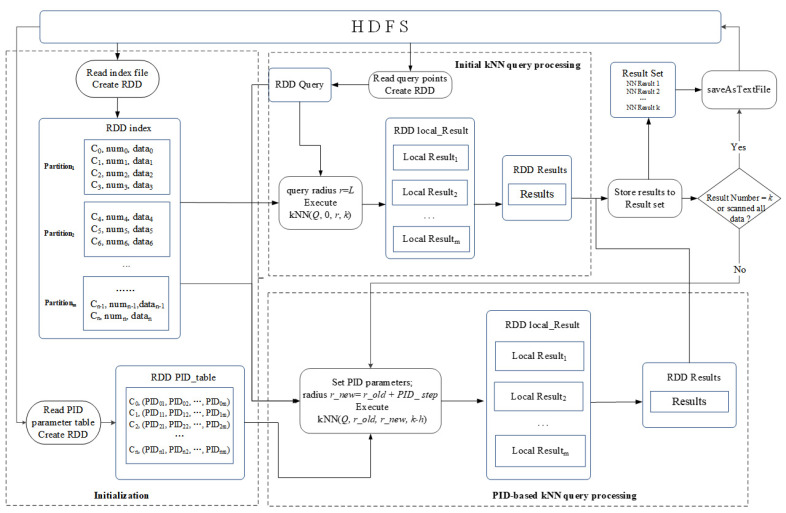
Overall framework of PIDKDD algorithm.

After the grid index is established, the data is clustered using a grid-based density peak clustering algorithm. Corresponding PID parameters are set for each data cluster through experiments. Thereby, the mapping table among grid cells, clusters, and the corresponding PID parameter values is established. On this basis, we designed a PID-based parallel kNN query algorithm (PIDKNN) based on the Spark platform. The PIDKNN algorithm mainly includes three stages: initialization, initial kNN query processing, and PID-based kNN query processing. The overall processing framework is shown in Figure 4.

(1) Initialization. In the initialization stage, the grid index and PID parameter table are read from the HDFS file system, and the corresponding RDDs are created to prepare for the kNN query processing in the next phase.

(2) Initial kNN query processing. In this stage, the length *L* of the grid cell is taken as the initial kNN query radius, and the initial kNN query is executed. The specific steps are as follows:

Step 1 reads the query point information from the HDFS system, calculates the corresponding grid cell where the query point is located according to the query point position information, and saves the grid cell ID and query point into the RDD Query.

Step 2 reads a query point *Q* from the RDD Query and then sets the query radius *r* = *L* (*L* is the length of the grid cell). For each data partition related to the query *Q*, a kNN query kNN(*Q, 0, r, k*) is executed, and a local kNN candidate result set RDD *Local_Result* is generated. The local kNN results of each partition are merged and sorted, and the first *k* results are stored in the result buffer *Result_set*.

Step 3 judges whether the number of results in the buffer *Result_set* meets the end condition; if so, the algorithm outputs *k* results in the buffer *Result_set* to the HDFS and ends the query. Otherwise, it proceeds to the next stage to execute PID_based kNN query processing.

(3) PID-based kNN query processing. In this stage, the difference between the current kNN query result number *h* and the target kNN result number *k* is calculated first, and then the PID parameter table PID_table is searched to obtain the corresponding PID parameter values according to the grid cell ID of the query point and query parameter *k*. The PID model is used to calculate the query step and generate a new query radius *r_new*. The kNN query kNN(*Q, r_old, r_new,* *k*-*h*) is performed on each data partition, and the local kNN candidate results are generated on each partition. The local kNN results of each partition are merged and sorted, and the first *k*-*h* results are saved in the buffer *Result_set*. After that, judging whether the number of results in the buffer is sufficient, if the requirements are met, the algorithm outputs *k* results from the buffer *Result_set* to HDFS and ends the query. Otherwise, it repeats (3) until the requirement is met or the whole data partition is searched.

The kNN query function kNN (*Q, r_old, r_new, k*) means: take query point *Q* as the center of the circle, draw circles with radius *r_old* and *r_new*, respectively, and execute kNN queries in the area between the two circles. The specific process is as follows:

(1) For any grid cell *C, mindist (C, Q)* is used to represent the minimum distance from query point *Q* to grid cell *C* and *maxdist (C, Q)* is used to represent the maximum distance from query point *Q* to grid cell *C*. If the conditions *mindist (C, Q) ≤ r_new* and *maxdist (C, Q) ≥ r_old* are satisfied, the region represented by grid cell *C* needs to be searched, and *C* is put into the candidate query queue.

(2) A grid cell is taken out from the candidate query queue. For each data point *p* in the grid cell, the distance *dist (p, Q)* between the data point *p* and the query point *Q* is calculated. If *r_old < dist(p, Q) ≤ r_new*, then the point *p* is put into the result set *R*. All data points in *R* are arranged in ascending order according to the distance from the query point *Q*. When the number of elements in *R* reaches *k*, the distance from the *kth* element to the query point *Q* is assigned to *r_new*, that is, *r_new* = *dist (Result_set [k], Q)*. The next grid cell is fetched from the candidate query queue and 2) is continued until there are no elements in the candidate query queue. The whole PIDKNN algorithm is described in Algorithm 1.
**Algorithm 1:** PIDKNN Algorithm**Input:** Spatial data object set: *DataSet*, query points: *Queryset*, grid cell length: *L*, the value of K: *k*, grid cell number: *num*

**Output:** kNN query result set  // Initialization  1. *Conf* = **new** SparkConf ( )  2. *sc* = **new** SparkContext (*conf*)  3. *Data* = *sc*.textFile (*DataSet*)  4. *ResultBuffer* = null  5. *Index* = Grid_index_build *(Data, num*)  6. *Cell_Cluster* = Grid_dense_peak clustering (*Index*)  7. *pid_list* = PID_build (*Cell_Cluster*)    //Initial kNN query processing  8. *Query* = *sc*.textFile (*QuerySet*)  9. *id* = getID (*query, Index*)  10. *r* = *L*
 11. *Local_Result* = kNN (*query, 0, r, k*)  12. *answer* = merged (*Local_Result*)  13. *answer* = *answer*.sort  14. *ResultBuffer*.save (*answer, k*)    // PID-based kNN query processing  15. **While** (*ResultBuffer*.length *< k* and *Queried_gridCells < num*)  16.   *sum* = *ResultBuffer*.length  17.   *pid_parameters* = get_pid_parameters (*k, id, pid_list*)  18.   *r_new* = *r* + PID (*k, sum, r, pid_parameters*)  19.   *h* = *ResultBuffer*.length  20.   *Local_Result* = kNN (*query, r, r_new, k- h*)  21.   *r = r_new*
 22.   *answer* = merged (*Local_Result*)  23.   *answer* = *answer*.sort  24.   *ResultBuffer*.save (*answer, k-h*)  25. **end while**
 26. *Result* = *ResultBuffer*.Saveastextfile (*k*)  27. **Return** *Result*
 28. *sc*.stop 
**def kNN** (***Query, r_old, r_new, k***):  29. gridCells_list = getIntersectingCells (*query, r_old, r_new*)  30. traversed_gridCells *= gridCells_list*.length  31. result = null  32. **for** (*gridCells_list: cell*)  33.   **for** (*cell: point*)  34.    **if** (*r_old < dist(Query, point)≤ r_new*) **then**
 35.     result.add(point)  36.    **end if**
 37. **end for**
 38. **Return** result

## 4. Experiments

In order to validate the effectiveness of our algorithm PIDKNN, we run a series of experiments on real and synthetic datasets and compare them to the existing methods SparkNN and ParallelCircletrip. This section will describe the specific experimental environment and the experimental results.

### 4.1. Experimental Environment

The experimental environment is a Spark cluster consisting of 5 IBM X3650 M4 servers, one of which is used as a Master and the rest as Workers. Each server is configured with an Intel Xeon E5-2620 CPU (6-core, 2.0 GHz), 32 GB DDR memory, and 6 TB hard disk. Each server is installed with CentOS 6.4 operating system, Hadoop 2.7.4 and spark-2.0-bin-hadoop2 cluster software, Scala and Java languages, and the corresponding algorithm modules.

### 4.2. Experimental Data

The proposed algorithm is tested by synthetic data sets and real data sets. The spatial distribution range of synthetic data sets is 10,000 × 10,000, and the spatial positions of data objects and query points are generated according to Gaussian distribution and uniform distribution, respectively. Ten sets of synthetic spatial data sets were generated in the experiment, and the number of data objects in each set was 0.5 million, 1 million, 2 million, 4 million, and 8 million. In the experiment, the number of Spark executors ranged from 6 to 72, and the value of kNN query parameter *k* ranged from 50 to 10,000. The real data set was GeoLite2, a global free IP address database, which collected about 5 million IP4 and IP6-related data points from 2020 to 2022. The details are shown in Table 1.

### 4.3. Experimental Results

Firstly, we performed ablation experiments on the PIDKNN algorithm by using synthetic data sets. Then, the performance of the PIDKNN algorithm was evaluated by using a synthetic data set and real data set. After that, we compared it with the ParallelCircularTrip algorithm and a recently proposed method under the Spark framework, SparkNN. The specific experimental results are given below.

#### 4.3.1. Ablation Experiments

**Figure 5:**
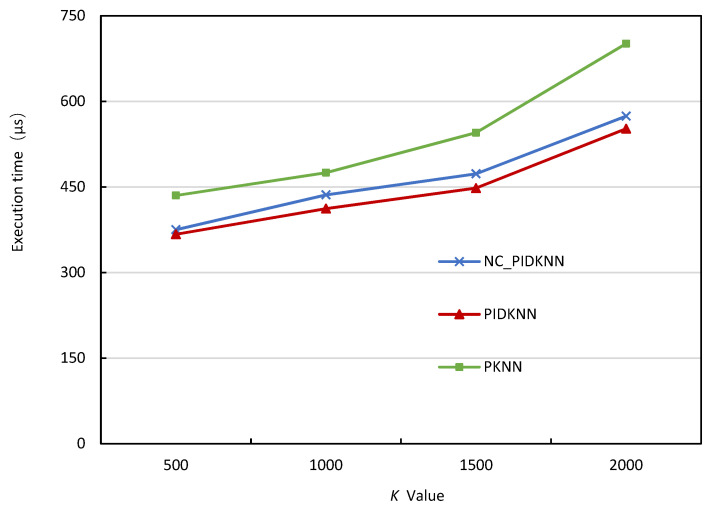
Execution time vs. *k* values.

Our approach, PIDKNN, mainly includes two parts: PID parameter setting based on density peak clustering algorithm and PID-based query radius calculation. In order to verify the effectiveness of these two parts, we conducted ablation experiments and constructed three algorithms, i.e., PKNN, NC-PIDKNN, and PIDKNN. PKNN is the traditional parallel kNN query algorithm that is based on a fixed query radius growth step. NC-PIDKNN is a parallel kNN query processing algorithm that uses PID-based query radius calculation and uniform PID parameter values for the whole data space. PIDKNN is our algorithm, which includes PID parameter setting based on density peak clustering algorithm and PID-based query radius calculation in two parts. Figure 5 shows how the execution time of the three algorithms changes with the value of *k* when the number of grid cells is 3600 and the data amount is 500,000. Figure 5 demonstrates that as the value of *k* increases, the execution time of the three algorithms also increases, consistent with expectations. It can be seen from Figure 5 that the execution time of PIDKNN and NC-PIDKNN algorithms is significantly lower than that of the PKNN algorithm. This is mainly because these two algorithms use PID to calculate the query radius and realize the query processing with a variable query radius growth step, which is superior to the traditional PKNN algorithm based on a fixed query radius growth step. This also shows that the PID-based query radius calculation proposed in this paper is very effective. At the same time, it can be seen that the execution time of PIDKNN is less than that of NC-PIDKNN, which also shows that using the density peak clustering method to set different PID parameter values for each data cluster is better than using unified PID parameter values for all data. It also shows that the PID parameter setting method based on the density peak clustering algorithm proposed in this paper is effective.

**Figure 6:**
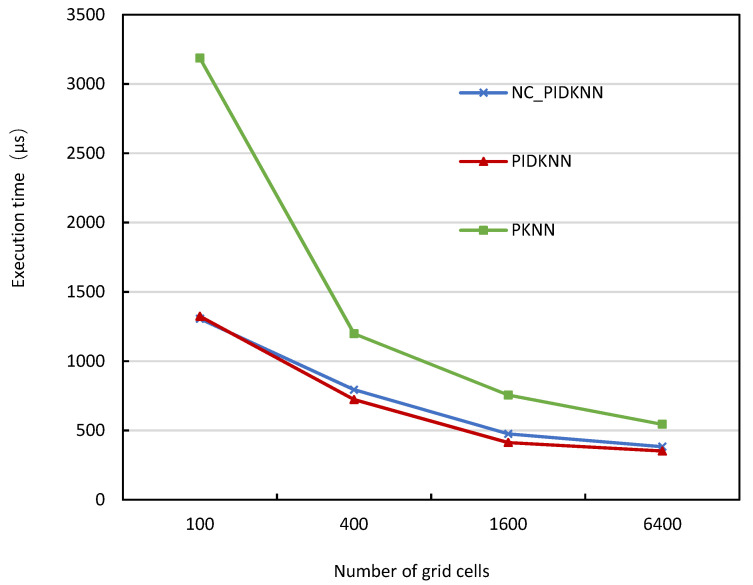
Variation of execution time with the number of grid cells for the three algorithms.

Figure 6 shows how the execution time of the three algorithms changes with the number of grid cells when the data size is 500,000 and the value of *k* is 2000. Figure 6 shows that as the number of grid cells increases, the execution times of the three algorithms decreases, which is as expected. In the beginning, the grid partition granularity is small, so that data skew creates an uneven data distribution, which, in turn, causes some tasks to take longer to run, and thus the overall execution time is relatively long. As the grid cells increases, data objects in the grid cells can be allocated to the executors more uniformly, and the running time thus decreases. It can also be seen from Figure 6 that the execution time of the NC_PIDKNN algorithm and the PIDKNN algorithm do not differ much and decrease with the increase in the number of grid cells. This is because the increase in the number of grid cells causes the data partition to become relatively more uniform, and the advantage of the PIDKNN algorithm is not obvious at this time. When the number of grid cells continues to increase, the parallel processing communication cost rises, which will lead to fewer benefits for the three algorithms, so the number of grid cells should be appropriate. From the experimental results of the above two aspects, it can be seen that the PIDKNN algorithm has the shortest execution time and is effective.

#### 4.3.2. Query Performance Comparison

In order to further verify the effectiveness of our algorithm PIDKNN, we first compare the PIDKNN algorithm and the ParallelCircularTrip algorithm. Both algorithms use grid index-based query processing. The main difference is that the ParallelCircularTrip algorithm uses a fixed query step growth, while PIDKNN adopts variable query step growth based on PID, so the grid-related parameter settings are all the same, which has good comparability. We first compare the execution times of the two algorithms from four different aspects of synthetic datasets. Among them, the execution time is the average value of randomly executing 100 kNN queries, and the execution time includes the index construction time.

1. Execution time comparison with different numbers of grid cells

**Figure 7:**
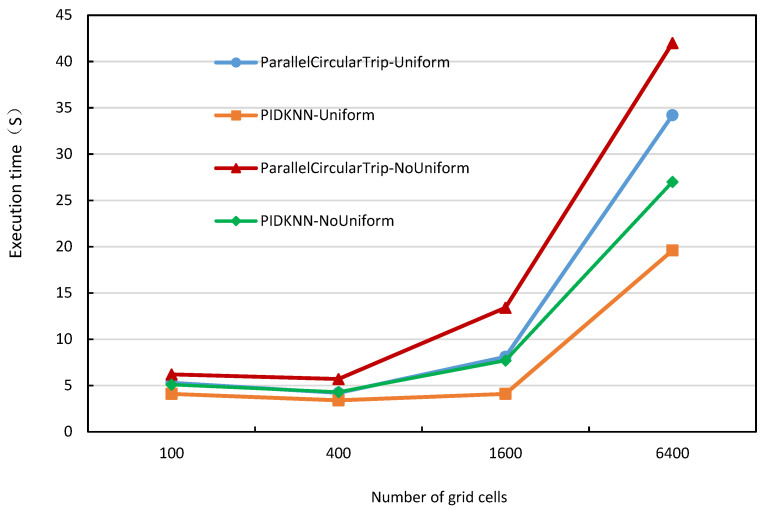
Execution time vs. number of grid cells.

For a fixed-size dataset, the number of grid cells directly affects the number of data objects in each cell and load balancing, so it has a significant impact on the performance of the algorithms. Figure 7 shows the execution time of the two algorithms with the number of grid cells when the dataset size is 2 million data objects, the *k* value is 10,000, and the number of Spark Executors is 12. From Figure 7, we can see that the execution time of the two algorithms first decreases and then slowly increases as the number of grid cells increases. This is because the increase in the number of grid cells reduces the number of data points in a single grid cell, the query involves more precise data points, and overall reduces the number of data points being queried, thus reducing the computation time. However, when the number of grid cells continues to increase, the amount of data involved in each grid cell continues to decrease, resulting in an increase in the search times to obtain a satisfied condition, which increases the execution time. At the same time, the execution time of both algorithms on non-uniformly distributed datasets is longer than that on uniform datasets. This is because the non-uniform data distribution makes the number of data objects in each grid cell vary greatly, resulting in inaccurate estimation of the kNN query radius, thus increasing the search time. From the test results on uniformly distributed datasets and non-uniformly distributed datasets, the PIDKNN algorithm is significantly better than the parallelcircularTrip algorithm, which shows that the PID-based query radius calculation is feasible and effective.

2. Execution time comparison with the size of data sets

**Figure 8:**
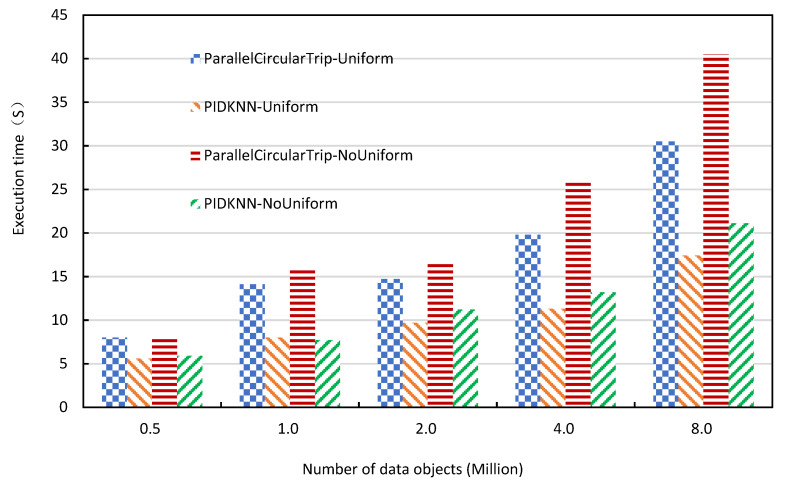
Execution time vs. number of data objects.

When the size of the data sets changes, the variation of the query execution time directly reflects the scalability of the algorithms. Figure 8 shows how the execution time of the two approaches changes with the data size of data sets when the *k* value is 10,000, the number of Spark executors is 24, and the number of grid cells is 6400. It can be seen from Figure 8 that the execution time of the two algorithms increases slowly with the increase of the data size on the uniform and non-uniform data sets, which is in line with expectations. At the same time, the increasing rate of execution time is relatively gentle, which shows that both algorithms have good scalability. The execution time of the two algorithms on non-uniform data sets is longer than that on uniform data sets. This is due to the non-uniform distribution of data sets, which makes the number of data objects in each grid cell vary greatly, resulting in the uneven distribution of the amount of computation allocated to each computing node, thus slowing down the overall completion time. From the experimental results on uniform and non-uniform data sets, the PIDKNN algorithm is significantly better than the ParallellcircularTrip algorithm, which further illustrates that the use of PID to predict the kNN query radius is useful, which reduces the number of searches and improves the efficiency of kNN queries.

3. Execution time comparison with the number of executors

**Figure 9:**
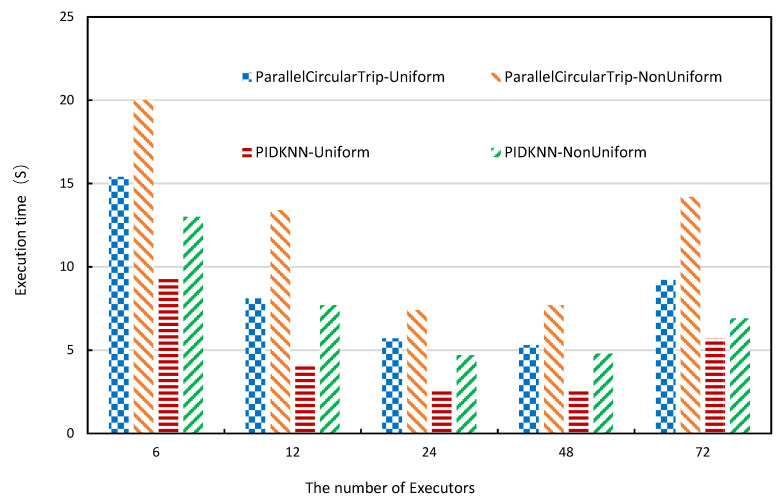
Execution time vs. the number of executors.

In the Spark system, the number of executors has a direct impact on the performance of the algorithm. A higher number of executors results in a higher degree of parallelism, which, in turn, should lead to faster execution time. Figure 9 shows how execution time changes with the number of executors over the synthetic data sets when the number of grid cells is 1600, the value of *k* is 10,000, and the size of the data sets is 2 million data objects. From the figure, it can be seen that the execution time of both algorithms gradually decreases as the number of executors increases and starts to rise after reaching a certain level, which is as expected. This is because the amount of computation on each executor decreases after the number of executors grows; thus, the execution time decreases, but when there are too many executors, it leads to more data exchange between executors, and the communication cost increases, thus affecting the query processing efficiency, which shows that for a fixed size data set, the number of executors should be appropriate. From the test results on both uniform and non-uniform data sets, the performance of the PIDKNN query processing algorithm is better than the ParallelCircularTrip algorithm.

4. Execution time comparison with different values of *k*

**Figure 10:**
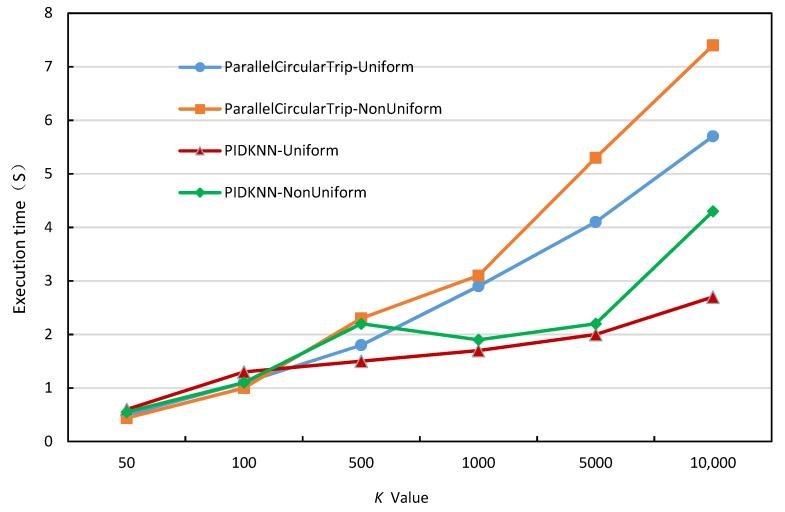
Execution time under different *k*.

As *k* is the main parameter in kNN query processing, execution times under different *k* values can directly reflect the scalability and robustness of the algorithms. Figure 10 shows the variation of the execution time of the two algorithms with the value of *k*, when the data set size is 2 million, a grid number of 1600, and the number of executors is 24. It can be seen from Figure 10 that the execution time of both algorithms on uniform and non-uniform data sets increases with the increase of *k* values. This is because when the value of *k* increases, the number of data objects to be searched increases, and the computational cost increases accordingly, resulting in an increase in the execution time of the two algorithms. When the value of *k* is small, there is no significant difference in the execution time of the two algorithms. This is because when the value of *k* is small, the kNN query requires only a few searches to obtain *k* results, and thus the query times of the two algorithms are close. However, when the value of *k* increases, the execution time of the PIDKNN algorithm is significantly lower than that of the ParallelCircularTrip algorithm, and the advantages are more and more obvious, whether it is on a uniform dataset or a non-uniform dataset. This is due to the fact that the PIDKNN algorithm introduces the PID control algorithm into the calculation of the query radius growth step, which optimizes the number of searches and reduces the total execution time. This further illustrates the effectiveness of the PIDKNN algorithm.

5. Comparison of PIDKNN and SparkNN

In order to further verify the effectiveness of the PIDKNN algorithm, we compared it with SparkNN, the latest kNN query processing algorithm for Spark. The test data comes from the real IP address data set of GeoLite2, with a quantity of 5 million. The average execution time of 100 queries is used as the performance measurement.

**Figure 11:**
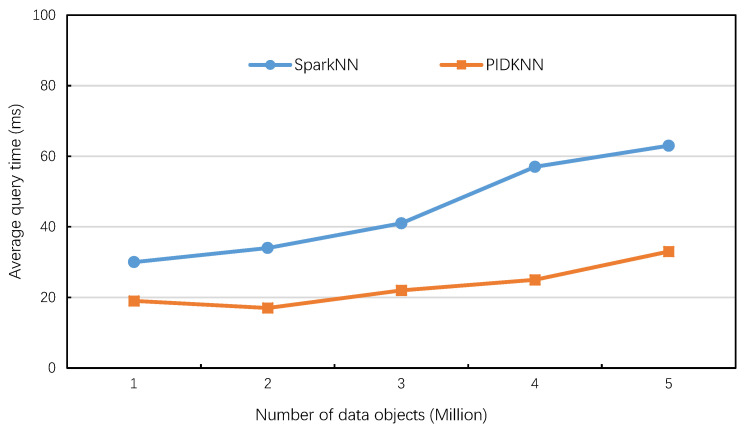
Average query time vs. number of data objects.

Figure 11 shows the variation in average query time of the two algorithms with the number of data objects when the value of *k* is 10 and the number of executors is 6. It can be seen from Figure 11 that the average query time of the two algorithms increases slowly with the increase of the data set, and the average query time of PIDKNN first decreases and then increases slowly. This is because when the number of grid cells remains the same, the increase of data objects leads to an increase of data objects in each grid cell, which helps to obtain *k* results as soon as possible, thus reducing the number of searches and reducing the total query time. However, as the number of data objects continues to increase, the number of objects involved in the query increases, resulting in a slow increase in query processing time. For the SparkNN algorithm, the increase of data objects leads to an increase in the depth of the KD-tree index, which leads to an increase in the index searching time, so the query execution time also increases slowly. Overall, the PIKDD algorithm is better than the SparkNN algorithm. The main reason is that the grid index adopted by our algorithm is simple and the search speed is fast. At the same time, the PID-based query radius calculation reduces the number of searches, thus showing better query performance. However, when the amount of data continues to increase, the grid needs to be further subdivided so that the data in each grid cell is limited to a certain range.

**Figure 12:**
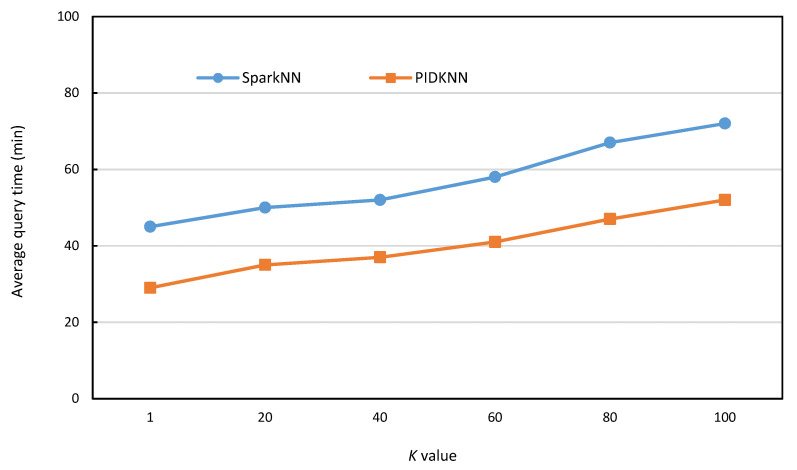
Average query time vs. *k* value.

Figure 12 is the comparison of the average query time of the two algorithms with different *k* values on a real data set with 5 million data. From the figure, it can be seen that the average query time of both algorithms increases slowly with the increase of *k* value, which is because the increase of *k* value increases the lookup range and thus the time increases. However, from the comparison of the two algorithms, the average query time of PIDKNN is better than that of SparkNN, mainly because PIDKNN uses variable step prediction based on the PID controller, which reduces the number of searches to a certain extent, thereby reducing the average query time. The SparkNN needs to look up the KD-tree index to find the data objects that satisfy the conditions, so its lookup efficiency is more stable and has better lookup efficiency for skewed data. From the comparison of the two algorithms, PIDKNN has certain advantages in query efficiency, but the PID parameter setting is relatively complicated.

## 5. Conclusions

kNN query processing on spatial big data has always been an important research topic in the field of spatial data management. We introduce PID controller technology into spatial kNN query processing and propose a PIDKNN algorithm based on the mainstream Spark big data processing platform to address the low efficiency of the existing kNN query algorithms in the distributed environment. The algorithm uses the grid partition method to realize the division of the entire spatial data and combines row sorting to realize data encoding and efficient indexing. The data is clustered by a grid-based density clustering algorithm, and different PID parameters are set for each type of data based on the sampling and experimental methods. On this basis, the PID controller algorithm is used to realize the kNN query processing with a variable query radius step based on the feedback mechanism, which greatly improves the efficiency of kNN query processing. A series of experimental results on synthetic data sets and real data sets show that the proposed PIDKNN algorithm has good performance and scalability and is significantly better than the existing parallel kNN query algorithms. The algorithm proposed in this paper still has some shortcomings. First, the sampling method is used to set the PID parameters through experiments, and the operation is relatively complicated. Second, the kNN query at the junction of different density data is not specially considered. Third, in the case of data skew, the efficiency of our algorithm has dropped significantly. These issues require further in-depth research in future work.

## Figures and Tables

**Table 1:** Experiential data parameters.

Parameter Name	Parameter Value
The spatial range of synthetic data	10,000 × 10,000
Data size of synthetic data sets (million) Size of real data set (million)	0.5 × 2, 1 × 2, 2 × 2, 4 × 2, 8 × 2 5
Parallelism (Spark Executors) Number of grid cells	6, 12, 24, 48, 72 100, 400, 1600, 6400
*K* Value	50, 100, 500, 1000, 5000, 10,000

## Data Availability

Publicly available datasets were analyzed in this study. This data can be found here: https://github.com/malyu/PIDKNN-Algorithm (accessed on 20 June 2022).
